# Flexible Iontronic Pressure Sensor Based on Ammonium Bicarbonate In-Situ Pore-Forming Porous Ionic Gel

**DOI:** 10.3390/mi17070787

**Published:** 2026-06-28

**Authors:** Zhiling Li, Zhixian Li, Liming Qin, Xiaodong Huang, Pan Pei

**Affiliations:** 1 Department of Intelligent and Information Engineering, Taiyuan University, Taiyuan 030032, China; 2024502001@tyu.edu.cn; 2 State Grid Xiangyuan County Electric Power Supply Company, Changzhi 046200, China; jixin-006@cpic.com; 3 Jinxi Industries Group Co., Ltd., Taiyuan 030027, China; 18803417630@163.com; 4 Key Laboratory of Instrumentation Science and Dynamic Measurement, North University of China, Taiyuan 030051, China; peipan@nuc.edu.cn

**Keywords:** flexible pressure sensor, iontronic sensor, porous ionic gel, in situ pore-forming, plasma-modified graphene electrode

## Abstract

To address prevalent industrial challenges, including the high cost of fabricating microstructures via photolithography and 3D printing, impurity residues easily generated by conventional physical/chemical pore-forming techniques, and the limited sensitivity of regular capacitive sensors, this paper innovatively proposes an integrated low-temperature in situ gas foaming strategy using ammonium bicarbonate for the fabrication of porous TPU-based ionic gels. Relying on the complete gaseous decomposition property of ammonium bicarbonate upon heating, a three-dimensionally interconnected continuous porous network is spontaneously constructed inside the polymer matrix. Thermoplastic polyurethane (TPU) is selected as the continuous polymer phase, and [EMIM][TFSI] imidazolium ionic liquid is blended as the ion source to synthesize composite ionic gel substrates. A PDMS composite slurry filled with graphene is employed to prepare flexible substrates, followed by low-temperature oxygen plasma surface modification to introduce polar functional groups such as hydroxyl and carboxyl onto electrode surfaces. A standard sandwich-structured ionic pressure sensor with the configuration of “top modified electrode—porous ionic gel dielectric layer—bottom modified electrode” is finally assembled. The porous framework and modified electrodes constitute a dual synergistic enhancement system: the porous structure markedly reduces the equivalent elastic modulus of the gel and improves its compressive deformation capacity; polar-modified electrodes optimize the interfacial compatibility between electrodes and gels, shorten ion migration paths and lower interfacial contact resistance. Systematic calibration of multiple batches of parallel samples reveals that the as-fabricated sensor achieves a high sensitivity of 25.3 kPa^−1^ across the full measuring range from 0 to 1000 kPa with a linear fitting coefficient R^2^ = 0.992. The loading response time and unloading recovery time of the device are 60 ms and 80 ms respectively, with a performance degradation of less than 3% after 1000 consecutive loading–unloading cycles, featuring low hysteresis error and excellent signal repeatability. Multi-scenario in vivo wearable tests on human subjects verify that the device can precisely capture subtle fluctuations of radial artery pulse and periodic laryngeal deformation during swallowing, distinguish characteristic waveform patterns of various English words according to differences in vocal cord vibration, and accurately detect bending motions when attached to finger joints. The entire fabrication process adopts common chemical raw materials and standard laboratory equipment without expensive micro-nano processing facilities, featuring convenient raw material procurement and high process fault tolerance, which enables large-area coating-based mass production. This work delivers a novel technical route for the low-cost large-scale production of high-performance ionic flexible sensors and bears significant industrialization reference value for applications in wearable medical monitoring, bionic robotic electronic skin, flexible human–machine interactive touch panels and other related fields.

## 1. Introduction

With the rapid iteration of new-generation artificial intelligence, wearable medical care and soft robotics technologies, flexible pressure sensing technologies have been regarded as a hotspot of interdisciplinary research spanning materials science, measurement and control technology, as well as biomedicine [1,2,3]. The global market scale of flexible sensors has been maintained at an annual growth rate of over 18% in the past five years, and various flexible pressure components have been extensively adopted in segmented application scenarios, including household health monitoring bracelets, tactile systems for intelligent prostheses, on-line detection of industrial curved surface deformation and human–machine flexible touch control [4,5,6]. Conventional metal strain gauges and ceramic pressure elements are featured by high hardness and poor ductility; hence, they cannot be closely attached to irregular human body surfaces and curved surfaces of special-shaped workpieces, and their applicable application scenarios are severely restricted [7]. Polymer-based flexible pressure sensors have gradually been developed into mainstream sensing components by virtue of their merits such as light weight, foldability, favorable biocompatibility and thin-film manufacturability [8]. Classified according to the force-electricity conversion mechanism, currently prevailing flexible pressure sensors are divided into four major technical routes, namely piezoresistive [9], conventional capacitive [10], piezoelectric [11] and triboelectric nanogenerator-based types [12]. Distinctions are obviously made in their application scopes, as discrepancies are presented in material selection, structural design, applicable measuring range, environmental resistance and manufacturing cost among the four types of devices. Piezoresistive flexible sensors were put into industrialization earliest; simple fabrication processes and low technical barriers for the design of post-processing acquisition circuits are possessed by such sensors, whose resistance variations are realized via the spacing change in conductive fillers under external force. Nevertheless, the polymer substrates of such devices are prone to creep drift induced by ambient temperature and humidity, severe baseline drift is generated after long-term static compression, and tiny resistance variations are triggered under ultra-weak physiological pressure, resulting in a low signal-to-noise ratio; accordingly, high-precision detection of ultra-faint signals such as arterial pulses cannot be accomplished [13,14,15]. Signal outputs of piezoelectric devices are achieved relying on polarized charges generated by piezoelectric polymers, yet only instantaneous dynamic impact loads can be responded to, and continuous collection of constant static pressure cannot be fulfilled, which makes such devices unsuitable for long-term continuous monitoring of human physiological signs [16,17,18]. Electrical potential outputs of triboelectric nanogenerators are obtained through interfacial triboelectrification, while output waveforms are extremely susceptible to disturbances from ambient humidity and contact surface roughness; severe signal distortion is caused under humid conditions, and their practicability is limited in the field of medical wearable devices [19,20,21]. Superiorities including simple configuration and ultralow power consumption, as well as outstanding static stability, are owned by traditional parallel-plate capacitive sensors, whereas capacitance variations are only achieved via bulk polarization of dielectric materials, limited electrical response amplitudes are produced after compression, and the sensitivity under low-pressure loading can hardly be improved, which is identified as the core bottleneck restricting their popularization in the field of high-precision physiological monitoring [22,23,24].

The iontronic sensing concept was formally put forward by Pan’s group in 2011 [25]. Large variations in capacitance can be induced by tiny deformations on the basis of the electric double-layer polarization effect at the electrode–ion gel interface, and the inherent low-sensitivity drawback of conventional capacitive sensors can therefore be well compensated. Targeted R&D efforts have been focused on iontronic sensors worldwide over the past decade. Ionic gels are fabricated via mixing and curing of polymer matrices with room-temperature ionic liquids. Free anions and cations contained inside can be rapidly migrated and accumulated toward interfaces under the combined action of external electric fields and mechanical loads, and ionic gels are recognized as the core sensitive medium of iontronic devices [26,27,28]. Current optimizations targeted at ionic gels are categorized into two primary approaches. On one hand, formulations of polymer matrices are modified, and the mechanical properties of materials are optimized through copolymerization and filler doping. On the other hand, microstructures are constructed on the surface or inside dielectric layers, and elastic moduli are reduced to improve deformability. It has been verified in numerous published literature that microstructure optimization is ranked among the most efficient strategies for the sensitivity enhancement of iontronic devices. Two typical configurations, including surface protrusion arrays and internal porous networks, are adopted for such microstructures [29,30,31].

In recent years, microstructures have been widely introduced into the dielectric layer design of pressure sensors for the improvement of sensor sensitivity. As early as 2010, elastic dielectric layers with pyramidal microstructures were adopted by the research team from Stanford University [32], and a remarkable enhancement in the sensitivity of flexible capacitive pressure sensors was achieved. Nevertheless, relatively complicated fabrication procedures are required for such microstructure-containing dielectric layers, and the overall manufacturing cost of sensors is raised accordingly. An ultrahigh-sensitivity flexible pressure sensor was reported by Xia et al. [33]. The device was constructed from a 3D-printed flexible substrate with hollow microstructures, gold films sprayed onto the substrate, and silver interdigital electrodes. An ultrahigh sensitivity of 419.622 kPa^−1^, a rapid response time of 30.76 ms, and a recovery time of 15.17 ms were exhibited within an ultralow pressure regime (<100 Pa). However, prohibitively expensive equipment is required for 3D printing technology, and mass production cannot be realized. A facile and scalable fabrication route for flexible pressure sensors with high sensitivity and broad detection range was developed by Du et al. [34] via the combination of silver nanowire coating, hierarchically microstructured PDMS fabricated by laser ablation, and interdigital electrodes. Similarly, mass production of sensors is restricted by the high cost of photolithography. It has been indicated by previous investigations that protruding microstructures on dielectric surfaces are mostly fabricated with photolithography, 3D printing or template replication for existing high-sensitivity sensors. Large capital investment in equipment and cumbersome customized mold procedures are involved in the above-mentioned techniques, so high costs are maintained for mass manufacturing. Meanwhile, irregular pore distribution and residual impurities are frequently induced by conventional particle-templated, pore-forming and freeze-drying pore-making methods. Therefore, the exploration of a low-cost, facile and mass-producible microstructure fabrication strategy is still regarded as a major challenge.

To address the above issues, this paper proposes a novel integrated strategy for fabricating TPU-based porous ionic gels via in situ low-temperature gas-phase foaming of ammonium bicarbonate. The fundamental distinctions between this technology and conventional foaming, particle-induced pore-forming and sacrificial template methods are as follows: For the first time, full gas-phase foaming of NH_4_HCO_3_ is introduced into the TPU-[EMIM]+[TFSI]- ionic gel system, simultaneously balancing the stability of ionic liquids and the continuity of polymer pore formation. The decomposition products of ammonium bicarbonate are only NH_3_, CO_2_ and H_2_O, which volatilize completely after heating without solid residues, thereby overcoming the drawbacks of traditional sacrificial pore-forming particles such as NaCl and sucrose that require water washing and tend to contaminate ionic liquids. Foaming, shaping and thin film formation are accomplished in one step, followed by oxygen plasma electrode interface modification to construct a dual synergistic enhancement system of “porous medium + polar interface”, while previous studies solely focusing on foaming failed to carry out collaborative interface optimization. The addition of gradient pore-forming agents and segmented temperature control processes suppresses the violent boiling of THF solvent and excessively rapid foaming, enabling precise regulation of three-dimensional interconnected pore networks while balancing mechanical resilience and ion transport efficiency. The entire process eliminates the need for expensive micro-nano processing equipment, features cheap and readily available raw materials, and supports large-area coating for mass production. The assembled ionic pressure sensors achieve wide-range and highly sensitive pressure detection, possessing industrialization potential in the fields of wearable medical devices and electronic skins.

## 2. Research on Fabrication and Technology of Flexible Pressure Sensor

### 2.1. Experimental Raw Materials and Detailed Parameters

Thermoplastic polyurethane TPU1185A (BASF industrial grade, Shore hardness 85A, number-average molecular weight 85,000, melting temperature 185 °C); 1-Ethyl-3-methylimidazolium bis(trifluoromethanesulfonyl)imide [EMIM][TFSI] (Lanzhou Institute of Chemical Physics, purity 99.5%, liquid at room temperature, ionic conductivity 4.2 mS/cm); N,N-Dimethylformamide DMF (Sinopharm analytical reagent grade, boiling point 153 °C); tetrahydrofuran THF (Sinopharm analytical reagent grade, boiling point 66 °C); ammonium bicarbonate NH_4_HCO_3_ (Kermel analytical reagent grade, initial decomposition temperature 36 °C, complete decomposition at 60 °C, prone to slow decomposition at ambient temperature, stored away from light at 4 °C); few-layer graphene (sheet diameter 5~15 μm, single-layer proportion 83%, carbon content ≥ 98%); PDMS (Dow Corning 184, base agent:curing agent = 10:1, crosslinking and curing at room temperature); PET film (thickness 0.1 mm, transverse tensile strength 180 MPa); oxygen-free copper wire (wire diameter 0.1 mm); anhydrous ethanol and deionized water.

### 2.2. Experimental Instruments and Process Parameters

Digital display constant-temperature magnetic stirrer (temperature control accuracy: ±0.3 °C, adjustable rotating speed ranging from 0 to 1500 r/min); numerical control ultrasonic cleaner (rated power: 300 W, frequency: 40 kHz); programmable desktop spin coater; electric blast drying oven (temperature control range: ambient temperature to 300 °C); PT-100 oxygen plasma processor (RF power: 0~300 W, chamber volume: 5 L); FA2004 electronic analytical balance (precision: 0.0001 g); micrometer thickness gauge, optical microscope and vacuum drying oven.

### 2.3. Standardized Pretreatment of Raw Materials

TPU pellets are vacuum-dried at 60 °C for 12 h to remove adsorbed moisture inside pellets and prevent caking during dissolution; ammonium bicarbonate is hermetically stored under refrigeration and weighed right before experiments to avoid premature decomposition and loss at ambient temperature; graphene powder is vacuum-dried at 80 °C for 8 h to eliminate residual solvent trapped within agglomerated powder gaps; PET and glass substrates are wiped with anhydrous ethanol followed by 15 min of ultrasonic cleaning and subsequent drying to remove grease, ensuring stable adhesion of coated films.

### 2.4. Exploration of Pre-Experiments on Formulation Gradient

The official formulation was screened via multi-gradient pre-experiments: DMF and THF were each fixed at 20 mL, TPU at 3.5 g and ionic liquid at 5 mL, with ammonium bicarbonate dosage set at four levels of 1 g, 2 g, 3 g and 4 g. At 1 g, insufficient foaming volume, sparse pores and inadequate compression space were found; at 2 g, relatively low porosity was found; at 3 g, well-connected pore structures without large-area pore collapse were found; at 4 g, excessive foaming accompanied by massive ruptured bubbles forming through-going large cavities and deteriorated mechanical properties of the gel was found. Three groups of control experiments with dissolution temperatures set at 55 °C, 65 °C and 75 °C were carried out. At 55 °C, the dissolution rate of TPU and the volatilization rate of solvent are slow, leading to an excessively long foaming cycle and low production efficiency. At 75 °C, THF is close to its boiling point and prone to violent bumping, which causes instantaneous rupture of bubbles and collapse of pore structures. Meanwhile, prolonged high-temperature treatment accelerates trace volatilization of ionic liquid on the surface and reduces the ionic conductivity of the gel. Preliminary experiments verified that the impedance of ionic gel increased by 17% after constant-temperature treatment at 75 °C for 2 h, so this temperature was eliminated. At 65 °C, balanced efficiency of TPU dissolution and uniform foaming of ammonium bicarbonate can be achieved; THF volatilizes slowly without bumping, and no volatilization loss of ionic liquid occurs. Thus, 65 °C is identified as the optimal foaming and film-forming temperature in this study.

### 2.5. Preparation of Dielectric Layers for Flexible Pressure Sensors

**Figure 1:**
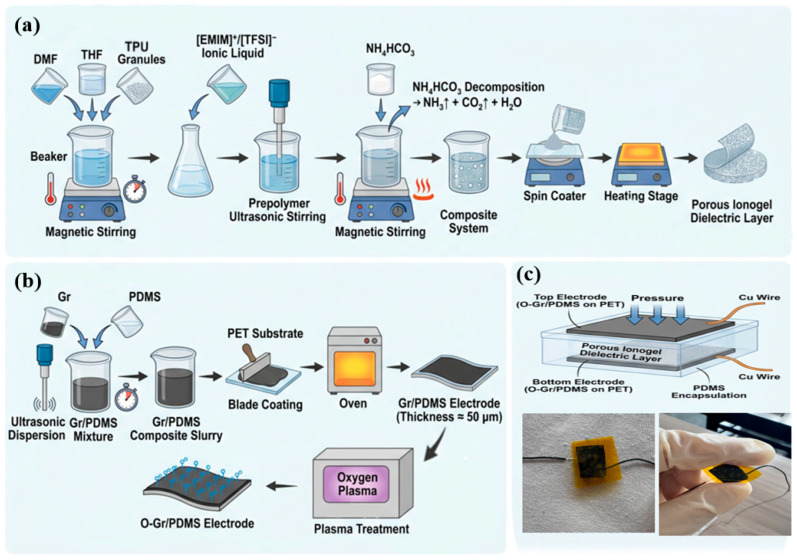
Fabrication of flexible pressure sensors: (**a**) Fabrication flow chart of the dielectric layer. (**b**) Fabrication flow chart of the electrode layer. (**c**) Sandwich structure of flexible pressure sensor and physical photograph of the complete device.

The preparation process of the dielectric layer for flexible pressure sensors is illustrated in Figure 1a. A mixed solvent consisting of equal volumes of DMF and THF (20 mL each) was prepared, into which 3.5 g of TPU pellets were added. The mixture was subjected to constant-temperature magnetic stirring at 65 °C for 5 h until the pellets fully swelled to form a transparent base solution. Subsequently, 5 mL of high-purity [EMIM]+[TFSI]- ionic liquid was incorporated, followed by ultrasonication at 65 °C for 2 h to achieve uniform dispersion of ions. Three grams of ammonium bicarbonate powder was added to the premixed solution, and staged low-temperature stirring with precise temperature control was adopted for foaming: the mixture was first stirred at a low speed at 50 °C for 10 min to uniformly disperse ammonium bicarbonate, then heated at a constant rate to 65 °C and held at this temperature for 25 min. Continuous bubble channels were generated in situ within the system via gaseous products (NH_3_, CO_2_ and H_2_O) released from the decomposition of the foaming agent. After the entire system was filled with pores, the solution was spin-coated onto a glass substrate, and the solvent was evaporated via gradient temperature ramping: the sample was maintained at 65 °C for 1 h to slowly remove most of the THF and DMF, then heated to 90 °C and held for 2 h to completely decompose residual ammonium bicarbonate and volatilize all remaining solvent, enabling physical shaping through entanglement of TPU molecular chains. After cooling, the film was peeled off from the glass substrate to obtain a porous ion gel dielectric layer with a thickness of 200 μm.

### 2.6. Preparation of Electrode Layer for Flexible Pressure Sensor

The preparation process of the electrode layer for the flexible pressure sensor is illustrated in Figure 1b. An amount of 300 mg of Gr is blended with 10 mL of PDMS and dispersed via ultrasonic treatment for 1 h to obtain Gr/PDMS composite slurry. The composite slurry is blade-coated onto a PET substrate, followed by pressing frosted glass against the coated slurry. After drying in an oven at 80 °C for 2 h, the frosted glass is peeled off to fabricate Gr/PDMS electrodes with a thickness of approximately 50 μm. An oxygen plasma processor (PT-100) is utilized to modify the electrode surface under a processing power of 100 W for 30 s to introduce oxygen-containing functional groups, and the modified electrode is designated as O-Gr/PDMS electrode, while the untreated counterpart is marked as Gr/PDMS electrode.

A four-probe sheet resistance tester was used to quantitatively characterize the electrical conductivity of electrodes before and after plasma treatment: the sheet resistance of the pristine Gr/PDMS electrode was 12.6 Ω/sp; after oxygen plasma treatment at 100 W for 30 s, the sheet resistance reached 13.1 Ω/sp, representing only a slight increase of 3.97%. The internal conductive interconnection network of graphene remained intact, and only a small amount of sp^2^ carbon on the electrode surface was mildly oxidized. The overall electrical conductivity fully meets the application requirements of flexible electrodes. Plasma modification only regulates surface chemical functional groups without damaging the intrinsic conductive pathways of the electrode.

### 2.7. Fabrication of Flexible Pressure Sensors

Porous ionic gel (10 mm in diameter and 200 μm in thickness) is adopted as the sensitive layer and sandwiched between two flexible electrodes. Copper wires are led out from the electrodes, and a small amount of PDMS is applied for peripheral encapsulation to obtain a separated flexible pressure sensor, whose structural schematic diagram is displayed in Figure 1c. The sensor size is 10 mm × 10 mm.

## 3. Working Mechanism and Characterization of Flexible Pressure Sensor

This device adopts a sandwich-type capacitive sensing structure, and its overall equivalent capacitance is formed by the coupling of two components: the geometric capacitance of parallel plates and the electric double-layer capacitance at the electrode/ionic gel interface. The expression for total equivalent capacitance is as follows:

(1) $$C_{total}=C_{geo}+C_{EDL}$$

Among them, the geometric parallel-plate capacitor:

(2) $$C_{geo}=\frac{\varepsilon_{0}\varepsilon_{r}S}{d}$$

C_EDL_ stands for the total capacitance of the electric double layer formed at the contact surfaces between the two electrodes and the ionic gel; ε_0_ = 8.854 × 10^−12^ F/m denotes the vacuum permittivity; ε_r_ represents the equivalent relative permittivity of the porous ionic gel; S refers to the actual effective bonding area between electrodes and the dielectric medium; d is the thickness of the dielectric sandwiched between the upper and lower plates.

To verify the rationality of this series superposition model, a wide-band impedance-capacitance sweep test ranging from 100 Hz to 10 kHz was carried out. In the low-frequency range (100–1000 Hz), the electric double-layer capacitance *C*_EDL_ dominates, and the capacitance varies drastically with pressure. In the high-frequency range (>5 kHz), the polarization response of the electric double layer lags behind, *C*_geo_ becomes the main contributing term, and the variation amplitude of capacitance decreases significantly. The frequency-domain test results directly confirm that the total capacitance of the device is formed by the coupling of geometric capacitance and interfacial electric double-layer capacitance, which validates the establishment of the model in Equation (1).

**Figure 2:**
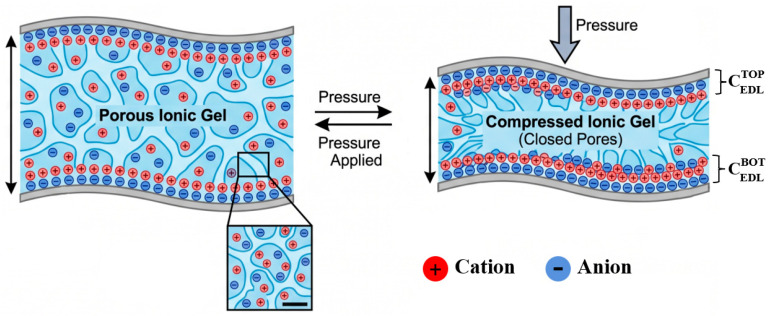
Working mechanism of flexible pressure sensor.

The operating principle of the pressure sensor is shown in Figure 2. In the initial state without external load, [EMIM]^+^ cations and [TFSI]^−^ anions inside the porous ionic gel are uniformly and randomly distributed. Under a weak applied alternating test electric field, ions only undergo random thermal motion, and a thermodynamically equilibrated electric double layer forms at the electrode-gel interface. The plate spacing d, effective contact area S and compactness of the dielectric remain constant, leading to fixed values of C_geo_ and C_EDL_, and the sensor retains its initial static capacitance C_0_.

When external pressure is applied perpendicularly to the top surface of the device, the three-dimensionally interconnected porous structure fabricated via in situ pore-forming undergoes elastic compression first. Different from the bulk deformation of dense non-porous media, numerous sealed cavities inside the porous framework collapse gradually, altering geometric capacitance parameters from two aspects. First, cavity collapse continuously reduces the overall medium thickness d. As indicated by Equation (2), *C*_geo_ is inversely proportional to *d*, so the reduced thickness directly increases the geometric capacitance. Second, trapped air inside pores is squeezed out, and the densification degree of the gel matrix is improved, which raises the equivalent dielectric constant *ε*_r_ of the composite material and further facilitates the growth of geometric capacitance. Meanwhile, the medium expands slightly in the transverse direction under compression, and the actual contact area S between electrodes and ionic gel keeps increasing, which also serves as a critical contributor to the rise of *C*_geo_.

Oxygen plasma modification is essential for enlarging the variation range of the electric double-layer capacitance. Untreated Gr/PDMS electrodes feature hydrophobic surfaces with large interfacial gaps and high ionic migration resistance. After oxygen plasma bombardment, abundant polar oxygen-containing functional groups, including hydroxyl (-OH) and carboxyl (-COOH), are generated on electrode surfaces. These functional groups improve electrode hydrophilicity to narrow interfacial gaps between electrodes and ionic gel on one hand, and adsorb surrounding free ions via electrostatic interaction to drastically lower the ionic migration barrier on the other hand. Driven by an external electric field, anions and cations inside the gel rapidly migrate directionally: cations accumulate near the negative electrode while anions concentrate around the positive electrode, accompanied by increased bound charges on electrode surfaces and compressed effective thickness of the electric double layer. According to the Helmholtz model of electric double layer, the decreased double-layer thickness and enlarged effective interfacial area both markedly boost *C*_EDL_. The synchronous and synergistic growth of geometric capacitance and electric double-layer capacitance ultimately leads to an obvious increase in total capacitance *C*_total_ with rising applied pressure.

The calculation formulas for relative capacitance change rate and sensor sensitivity are defined:

(3) $$S=\frac{\Delta C/C0}{\Delta P}=\frac{(C-C0)/C0}{\Delta P}$$

In the formula, C denotes the total capacitance after compression, C_0_ represents the initial no-load capacitance, ∆*P* stands for the pressure variation, and S is the sensor sensitivity with the unit of kPa^−1^.

After the external pressure is completely removed, relying on the excellent high elastic resilience of TPU polymer, the collapsed porous cavities recover gradually; the dielectric thickness d and electrode bonding area S return to their original dimensions, and ε_r_ reverts to its initial value. The anions and cations accumulated at the interface break free from electrostatic confinement and diffuse back into the bulk of the gel; the electric double layer regains its equilibrium state, C_EDL_ drops back to the initial level, and the total capacitance reversibly recovers to C_0_, accomplishing a single reversible conversion between pressure and electrical signal. The outstanding interfacial adhesion brought by modified electrodes effectively restrains interfacial delamination and debonding after numerous cycles, ensuring the long-term cycling stability of the device.

**Figure 3:**
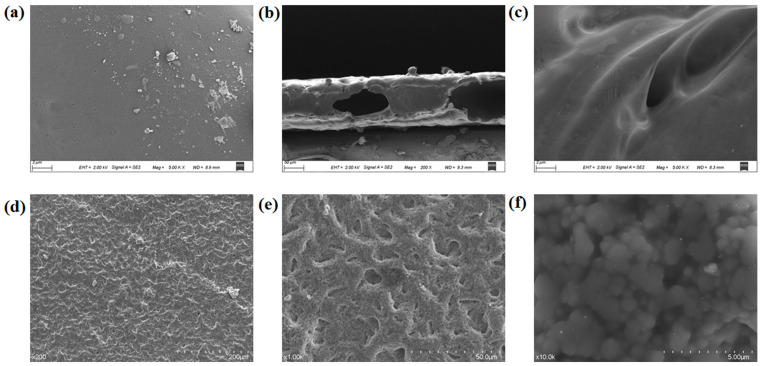
Characterization of flexible pressure sensors: (**a**) SEM image of the porous ionogel surface (scale bar: 2 μm, magnification: 5000×); (**b**) low-magnification SEM image of the ionogel cross-section (scale bar: 50 μm, magnification: 200×); (**c**) high-magnification SEM image of interconnected channels on the ionogel cross-section (scale bar: 2 μm, magnification: 5000×); (**d**) low-magnification surface morphology of Gr/PDMS electrode (scale bar: 200 μm, magnification: 200×); (**e**) medium-magnification dispersion morphology of graphene in Gr/PDMS electrode (scale bar: 50 μm, magnification: 1000×); (**f**) high-magnification microstructure of Gr nanosheets (scale bar: 5 μm, magnification: 10,000×).

The surface and cross-sectional micromorphologies of porous ionic gels were systematically characterized via field-emission scanning electron microscopy (FE-SEM), and the corresponding results are presented in the figures. As illustrated in Figure 3a, abundant, uniformly distributed micropores at the submicron scale exist on the ionic gel surface. These micropores originate from gas release during the thermal decomposition of NH_4_HCO_3_ and furnish initial compressible voids for the dielectric layer. Cross-sectional images in Figure 3b distinctly demonstrate an internally formed three-dimensionally interconnected porous network within the gel, with pore diameters ranging from several micrometers to tens of micrometers. The intact and continuous pore walls exhibit no evident collapse or fracture, verifying that the TPU matrix retains favorable structural stability under the combined effects of ionic liquid and pore-forming agent. The three-dimensional porous architecture fabricated via an in situ pore-forming technique effectively reduces the elastic modulus of ionic gels to facilitate deformation under external loading, while affording ample accommodation space for ionic liquids and enabling efficient ion transportation. Moreover, the large interconnected cavities observed from cross-sectional SEM images preferentially collapse under compressive stress to enable rapid detection of pressure signals, which well matches the outstanding sensitivity and dynamic response performance of the as-fabricated sensors in subsequent tests. The sample is pressed under 1000 kPa, the pressure is unloaded to 0 kPa, and cross-sections are cut for FE-SEM observation. After compression at 1000 kPa, the pores collapse under pressure and the pore channels close. After complete unloading and standing for 30 s, the pore size and interconnected structure of cross-sections are basically consistent with those of the original uncompressed sample, with no permanent fractures or irreversibly compacted regions. This directly verifies that the porous TPU skeleton features fully elastic and reversible rebound without irreversible pore collapse, as shown in Figure 3c.

The micromorphology of Gr/PDMS composite electrodes was characterized using field-emission scanning electron microscopy. Figure 3d reveals uniformly distributed micro-wrinkles across the electrode surface, which enlarge the specific surface area and facilitate sufficient interfacial contact with the ionic gel dielectric layer. Figure 3e further illustrates the dispersion state of graphene sheets within the PDMS matrix; overlapping graphene laminates construct uninterrupted conductive networks without severe agglomeration, confirming that ultrasonic dispersion achieves homogeneous dispersion of graphene inside PDMS and establishes stable conductive pathways for electrodes. Figure 3f explicitly displays the microscopic configuration of graphene laminates, which are embedded into the PDMS matrix with partial sheets exposed on the electrode surface to supply active sites for oxygen plasma modification. The homogeneously dispersed graphene conductive network paired with micro-rough surface morphology endows electrodes with superior electrical conductivity and mechanical robustness, and lays a solid structural foundation for subsequent oxygen plasma treatment. Post-modification electrodes possess remarkably improved surface hydrophilicity, which strengthens interfacial interactions with the ionic gel dielectric layer, reduces interfacial contact resistance effectively, and accordingly improves the response sensitivity and operational stability of ionic-type iontronic sensors.

## 4. Performance Testing of Flexible Pressure Sensor

**Figure 4:**
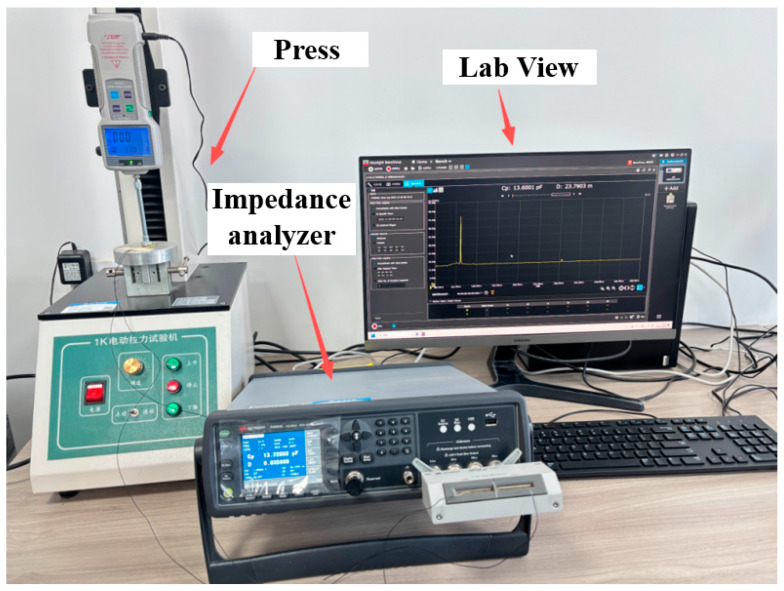
Testing system of flexible pressure sensor.

The flexible pressure sensor testing system is illustrated in Figure 4, consisting of a high-precision pressure tester, an impedance analyzer and a LabView upper computer data terminal. Uniform test conditions are set as follows: ambient temperature of 25 ± 2 °C and relative ambient humidity of 45 ± 3%RH; the pressure tester performs quasi-static compression at a constant loading rate of 0.5 mm/min to avoid dynamic impact. Five parallel specimens are fabricated for each group, and each group is tested three times, with the average value adopted to eliminate discrete fabrication errors. All specimens are placed statically for 24 h prior to testing to release internal packaging stress. The impedance analyzer is set with an alternating excitation voltage of 1 V and a test frequency of 1 kHz, with all testing parameters kept constant. The sensor is attached to the pressure platform, followed by pressure loading via the pressure tester. The capacitance values corresponding to applied pressure are recorded by the impedance analyzer and further analyzed on the upper computer data terminal to obtain the characteristic performance curves of the sensor.

**Figure 5:**
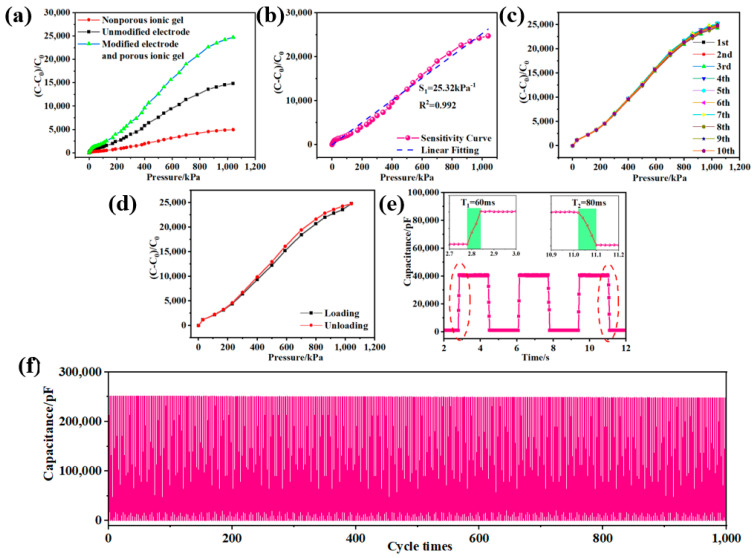
Performance testing of flexible pressure sensor: (**a**) sensitivity comparison of different devices; (**b**) sensitivity curve of the proposed sensor; (**c**) repeatability testing; (**d**) hysteresis testing; (**e**) response-recovery time; (**f**) long-term durability.

Three groups of control samples, including non-porous gel devices, devices with conventional unmodified electrodes, and devices equipped with porous gel plus modified electrodes, were fabricated separately to quantitatively compare the performance improvements originating from pore structure construction and electrode modification, as presented in Figure 5a. The experimental results reveal that sensors utilizing non-microstructured ionic gel dielectric layers deliver weak capacitance variation, with a relative capacitance change of approximately 5000 under 1000 kPa pressure and correspondingly low sensitivity. After the introduction of porous ionic gel, the device sensitivity rises remarkably, achieving a relative capacitance change of around 15,000 at the identical applied pressure. The combination of porous ionic gel and oxygen plasma-modified electrodes brings about a dramatic enhancement in device response performance; the relative capacitance change reaches up to 25,000 under 1000 kPa pressure, vastly exceeding the values of the other two control devices. These findings demonstrate that the porous structure improves compressibility by reducing the elastic modulus of the dielectric layer, whereas electrodes modified via oxygen plasma further amplify the responsive signal of ionic–electronic double-layer capacitance by strengthening interfacial contact and cutting down contact resistance. The synergistic effect of these two factors constitutes the core prerequisite for realizing high sensing sensitivity.

Figure 5b plots the relative capacitance variation curve of the sensor within the pressure range of 0–1000 kPa. It can be observed that the relative capacitance of the sensor ((C-C_0_)/C_0_) rises in an approximately linear manner with increasing pressure, with a linear fitting coefficient R^2^ = 0.992, demonstrating the outstanding linear response performance of the prepared sensor. Calculation results reveal that the sensor achieves a sensitivity of 25.3 kPa^−1^, which is remarkably superior to that of numerous conventional capacitive pressure sensors. The ultrahigh sensitivity originates from the synergistic effect of the low-modulus property of porous ionic gel and the iontronic sensing mechanism. Upon pressurization, the porous structure collapses rapidly to reduce the thickness of the dielectric layer and enlarge the contact area between electrodes and the dielectric layer simultaneously; these two factors jointly trigger a sharp rise in electric double-layer capacitance.

Ten consecutive loading–unloading cyclic tests were carried out to evaluate the repeatability of the sensor, and the results are presented in Figure 5c. The response curves from the ten cycles nearly overlap completely without obvious baseline drift or performance degradation, demonstrating the favorable repeatability of the sensor. Figure 5d displays the hysteresis curves during loading and unloading. The two curves match closely with a low hysteresis error, verifying that the porous ionic gel possesses outstanding elastic recovery capacity and can rapidly revert to its original shape after external force is removed, which is critical for the long-term stable operation of the sensor.

Response speed is an important indicator for evaluating the dynamic performance of sensors. Figure 5e illustrates the response and recovery process of the sensor under applied pressure. Upon pressure loading, the capacitance of the sensor rises rapidly to a steady value within 60 ms; after pressure removal, the capacitance returns to its initial state within 80 ms. Such fast response and recovery characteristics are mainly ascribed to the low elastic modulus of the porous ionic gel and favorable interfacial contact between the electrode and dielectric layer, enabling the sensor to promptly track variations in external pressure.

To further verify the long-term durability of the sensor, 1000 consecutive pressure loading–unloading cyclic tests were carried out on the sensor, as depicted in Figure 5f. Throughout the entire testing process, the capacitive response signal of the sensor remained stable with a performance degradation of less than 3%, demonstrating that the device possesses outstanding long-term mechanical stability and fatigue resistance and can meet the requirements for long-term application in wearable devices.

## 5. Practical Application Testing of Flexible Pressure Sensor

**Figure 6:**
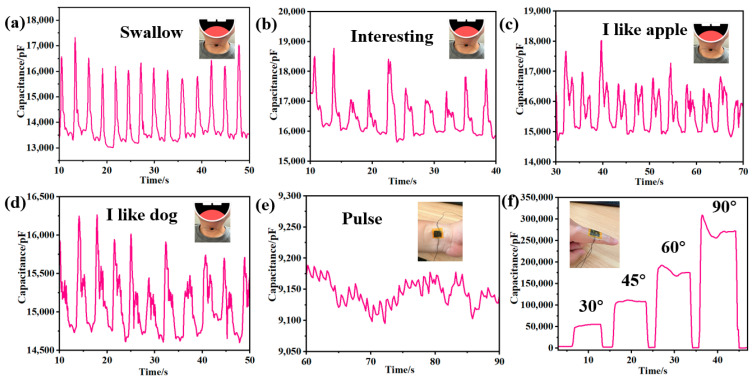
Practical application tests of flexible pressure sensors: (**a**) swallowing test of the pressure sensor; (**b**) vocalization test of the pressure sensor for the word “interesting”; (**c**) vocalization test of the pressure sensor for the sentence “I like apple”; (**d**) vocalization test of the pressure sensor for the sentence “I like dog”; (**e**) pulse test of the pressure sensor; (**f**) finger bending test of the pressure sensor.

Thanks to its high sensitivity and fast response characteristics, this sensor can accurately capture weak human physiological signals. Figure 6a shows the swallowing motion signals recorded when the sensor is attached to the throat, which clearly present periodic waveform changes in swallowing pressure. In addition, the sensor can also be used for monitoring laryngeal vibration signals. As shown in Figure 6b–d, laryngeal vibration signals generated when pronouncing different words or sentences (such as “interesting”, “I like apple” and “I like dog”) possess distinctive characteristic waveforms. Machine learning classification algorithms will be constructed, and recognition accuracy statistics will be collected in follow-up research, which is expected to be applied in non-contact voice interaction systems. Figure 6e displays the pulse signals collected at the wrist; distinct pulse waveforms can be distinguished, enabling heart rate monitoring and cardiovascular health assessment.

The flexibility of the sensor enables it to closely attach to the surface of human joints and realize the monitoring of joint bending angles. Figure 6f presents the response signals of the sensor when the finger joint is bent at various angles (30°, 45°, 60°, 90°). As the bending angle increases, the pressure exerted on the sensor rises, and the capacitance value increases accordingly. In addition, the signals acquired at different angles can be distinctly differentiated, which demonstrates the promising application potential of the sensor in motion capture and rehabilitation training monitoring.

## 6. Quantitative Comparison of Microstructure Fabrication Processes

To highlight the advantages of the NH_4_HCO_3_ in situ pore-forming method in terms of low cost, scalability, and simplicity, it is systematically compared with mainstream techniques (Table 1). Soft templating, lithography, and 3D printing produce regular microstructures but rely on expensive equipment and complex processes, limiting mass production. Particle porogen and phase separation methods are low-cost but suffer from uneven pore size and poor controllability. Freeze-drying yields interconnected pores but requires long processing times and high energy consumption.

In contrast, the NH_4_HCO_3_ in situ pore-forming method, as a particle porogen strategy, offers ultra-low cost, simple process, no expensive equipment, and batch fabrication. NH_4_HCO_3_ decomposes completely into gases without solid residues, avoiding impurity issues. Pore size and porosity are easily tuned via porogen content and heating parameters. It is compatible with flexible matrices (TPU, PDMS) for large-area dielectric layer fabrication, demonstrating outstanding advantages in scalable flexible electronics manufacturing.

To intuitively highlight the comprehensive advantages of the ammonium bicarbonate in situ pore-forming iontronic pressure sensor fabricated in this work in terms of detection range, linear sensitivity, dynamic response, hysteresis and cycling durability, four typical literature works on flexible pressure sensors published in recent years are selected for horizontal quantitative comparison, covering three mainstream sensing systems including traditional capacitive, piezoresistive and iontronic types. All performance indicators are summarized in Table 2.

The comparison results in Table 2 demonstrate the following: The capacitive sensor reported in Reference [33] is constructed via 3D printed hollow microstructures, which only achieves ultrahigh sensitivity within an extremely narrow low-pressure range of 0–100 Pa, yet features an excessively limited detection range that fails to meet common wearable monitoring scenarios such as human joint movement and moderate external force detection. Meanwhile, its hysteresis and long-term cycling durability data are not provided. The piezoresistive sensor with laser-etched hierarchical microstructures in Reference [34] has an upper detection limit of merely 65 kPa, with its sensitivity drastically declining as pressure rises, and complete response–recovery curves as well as quantitative cyclic attenuation data are absent. The double-layer microstructure iontronic device in Reference [38] delivers fast response speed, but suffers a huge disparity in sensitivity across different pressure segments with drastically degraded performance under high pressure, and no quantitative cyclic attenuation values are available. The iontronic sensor with microstructured electrodes in Reference [39] exhibits overall low sensitivity across a wide measurement range and obvious hysteresis.

Compared with the above-reported sensors, the sensor fabricated in this work possesses remarkable comprehensive superiorities. First, its detection range is extended to 0–1000 kPa, covering multiple application scenarios from weak physiological signals to moderate external force stimulation. Second, it maintains a uniform sensitivity of 25.3 kPa^−1^ throughout the entire pressure range without segmented attenuation and exhibits outstanding linearity. Third, it achieves low hysteresis error, benefiting from the excellent resilience of the porous three-dimensional TPU framework. Fourth, its performance attenuation is less than 3% after 1000 loading–unloading cycles, guaranteeing reliable stability for long-term service. In addition, the entire in situ foaming process eliminates high-cost micro-nano fabrication equipment such as photolithography and 3D printing, making it more suitable for large-area mass production.

## 7. Conclusions

A high-sensitivity iontronic flexible pressure sensor based on a porous ionogel dielectric layer is successfully fabricated. The porous ionogel with low elastic modulus is prepared via the NH_4_HCO_3_ in situ pore-forming method, and a high-performance iontronic sensor is constructed with oxygen plasma-modified Gr/PDMS flexible electrodes. Comparative experiments confirm that the synergistic effect of the porous structure and electrode modification significantly enhances device performance. The sensor exhibits a sensitivity of 25.3 kPa^−1^, excellent linearity (R^2^ = 0.992), fast response/recovery time (60 ms/80 ms), low hysteresis, and outstanding long-term cycling stability (attenuation < 3% after 1000 cycles) within 0–1000 kPa. Benefiting from superior comprehensive performance, the sensor accurately detects physiological signals such as pulse and swallowing, and enables speech recognition and joint motion monitoring. This work provides a simple and effective fabrication method for high-performance multifunctional iontronic flexible pressure sensors, advancing their practical applications in wearable health monitoring and human–machine interaction.

## Figures and Tables

**Table 1:** **Comparison of different microstructure fabrication** **methods.**

Fabrication Method	Microstructure Type	Typical Pore/Feature Size	Cost Level	Scalability	Residual Impurities	Core Advantages	Key Drawbacks	Reference
Soft template method	Micro-pillar/micro-dimple array	10–100 μm	Medium-low	High	None	Well-defined microstructure, reusable template	High cost for high-precision master mold	[32]
Inorganic salt particle templating	Disordered porous structure	5–50 μm	Extremely low	Extremely high	Yes (particles require water washing for removal)	Simple operation, tunable porosity	Water washing contaminates ionogel, uneven pore size	[35]
Freeze-drying method	Hierarchical interconnected pores	1–100 μm	Medium	Medium-high	None	Excellent pore connectivity	Long preparation cycle (12–48 h), high energy consumption	[36]
UV photolithography	Precision micro-nano array	1–20 μm	Extremely high	Medium	Photoresist residue	Ultra-high pattern resolution	Expensive equipment, poor compatibility with flexible substrates	[34]
3D printing molding	3D gradient structure	20–200 μm	Medium-high	Medium	None	High structural design freedom	Slow printing speed, limited large-area fabrication	[33]
Phase separation method	Nanoporous structure	0.1–5 μm	Low	Extremely high	None	No additional porogen required	Poor pore size controllable, narrow process window	[37]
NH_4_HCO_3_ in situ gas foaming (This work)	3D interconnected porous network	1–80 μm	Extremely low	Extremely high	None	One-step film formation, large-area coating, adjustable porosity, compatible with ionogel	Moderately broad pore size distribution, mediocre structural order	This work

**Table 2:** **Comparison of Key Performance Between This Work and Reported Flexible Pressure** **Sensors.**

Reference	Sensing Mechanism	Detection Range	Sensitivity/kPa^−1^	Response/Recovery Time/ms	Hysteresis Characteristic	Cyclic Attenuation
[33]	Capacitive	0~100 Pa	419.622 (ultra-low pressure range)	30.76/15.17	Not reported	Not reported
[34]	Piezoresistive	0–65 kPa, minimum detection limit 3 Pa	0–22 kPa: 4.48 27–65 kPa: 0.86	Not reported	Good cycling stability	Good cycling stability
[38]	Iontronic	0–760 kPa	0–1 kPa: 51.8 1–760 kPa: 5.42	40/40	Small deviation between loading and unloading curves	No quantitative attenuation data
[39]	Iontronic	0–850 kPa	0–20 kPa: 2.0365 20–850 kPa: 1.9635	100/50	Slight hysteresis exists	2.79%
This work	Iontronic	0–1000 kPa	25.3 (uniform high linearity over full range)	60/80	Minor hysteresis	<3%

## Data Availability

The data presented in this study are available in this article.
